# Progesterone reduces cell survival in primary cultures of endometrioid ovarian cancer

**DOI:** 10.1186/s13048-019-0486-4

**Published:** 2019-02-08

**Authors:** Enrique Pedernera, María J. Gómora, Flavia Morales-Vásquez, Delia Pérez-Montiel, Carmen Mendez

**Affiliations:** 1 Departamento de Embriología, Facultad de Medicina, Universidad Nacional Autónoma de México, 04510 Ciudad de México, Mexico; 2 Departamento de Oncología Médica, Instituto Nacional de Cancerología, Secretaría de Salud de México, Ciudad de México, Mexico; 3 Departamento de Patología, Instituto Nacional de Cancerología, Secretaría de Salud de México, Ciudad de México, Mexico

**Keywords:** Ovarian cancer, Steroid hormones, Progesterone, Cell survival, Primary cell culture, Endometrioid ovarian carcinoma

## Abstract

**Background:**

Ovarian cancer is the most lethal of all gynecologic malignancies. The relationship between sexual steroids receptors and ovarian cancer progression has been largely evaluated. The presence of progesterone receptors has been associated with an increase of a disease-free period and overall survival in patients with ovarian carcinoma. In the present study, primary cultures of ovarian carcinoma obtained from 35 patients diagnosed with epithelial ovarian cancer were evaluated for cell survival after treatment with 10^− 8^ M of 17β-estradiol, progesterone, testosterone and dihydrotestosterone.

**Results:**

The results were analyzed considering histological subtypes: low grade serous, high grade serous, endometrioid and mucinous carcinoma; clear cell carcinoma was not included due to failure in obtaining successful cultures of this subtype. A significant reduction of cell survival was observed after progesterone treatment in endometrioid ovarian carcinoma. Changes were not observed in low grade serous, high grade serous and mucinous carcinoma. The effect of progesterone was related to the presence of progesterone receptor (PR), a 43% reduction in the cell number was observed in PR (+) endometrioid ovarian carcinoma.

**Conclusions:**

This study supports the importance of progesterone and the presence of progesterone receptor in the reduction of ovarian cancer progression in the endometrioid ovarian carcinoma.

## Background

Prevention of known risk factors and early detection have made possible to reduce breast and cervical cancer as a cause of death among women, although this is not the case with ovarian cancer diagnosis which is usually established at advanced stages [1–3]. Transvaginal ultrasound and CA125 blood determination, although useful for early detection, are not applicable to the whole population [4–6]. In industrial countries, ovarian cancer is the most lethal of gynecologal cancer and it is estimated that 185,000 women around the world will die from this disease in 2018 [7].

The relationship between sexual steroids receptors and ovarian cancer progression has been extensively studied [8–11]. A multicenter study provides evidence of a relationship between the presence of progesterone and estrogen receptors with overall survival in ovarian cancer patients; progesterone receptor has been associated to better prognosis in endometrioid and high grade serous carcinoma and estrogen receptor with higher overall survival in endometrioid carcinoma [12]. These results are similar to those of other studies that evaluate the association of progesterone receptor with an increase of disease-free period and overall survival [13–15]. Results observed with estrogen receptor and androgen receptor are less evident and remains controversial [9, 16–19].

The effect of estrogens and progesterone treatment has been evaluated in immortalized cell lines of ovarian cancer, 17β-estradiol stimulates cell proliferation and induces changes in gene expression [20], progesterone displays both stimulatory and inhibitory effects in the proliferation of cultured malignant ovarian cells in a dose-depending manner [21], and progesterone regulates gene expression and induces apoptosis in ovarian tumor cells [22–24]. On the other hand, only a few studies have evaluated the response to steroid hormone treatment in tumor explants incubation [25] or in a primary culture model [26], stressing the importance of carrying out further studies on the effects of sexual steroid hormones in ovarian carcinoma cells, in order to evaluate the individualized response of patients.

In the present study, primary cultures of ovarian carcinoma cells obtained from 35 patients diagnosed with epithelial ovarian cancer were evaluated for cell survival after treatment with 17β-estradiol (E_2_), progesterone (P_4_), testosterone (T) and dihydrotestosterone (DHT). The results were analyzed considering histological subtypes: low grade serous (LGSC), high grade serous (HGSC), endometrioid and mucinous carcinoma; clear cells carcinoma was not included due to a failure in obtaining successful cultures of this subtype. A significant reduction of cell survival was observed with progesterone treatment in endometrioid ovarian carcinoma.

## Methods

In this prospective study we evaluated the total number of cells recovered from primary cultures of ovarian tumors after treatment with 17β-estradiol, progesterone, testosterone and dihydrotestosterone. Samples of ovarian carcinoma (*n* = 93) were obtained from patients of the Instituto Nacional de Cancerología(INCan) in Mexico City undergoing initial laparotomy, without any previous treatment and confirmed diagnosis of primary ovarian carcinoma, the exclusion criteria applied for the samples being a different diagnosis from primary ovarian carcinoma and a refusal by patients to sign the participation consent form. The National Institute of Cancerology is a tertiary referral hospital that receives adult patients from the central region of Mexico. The study protocol was approved by the Ethic Committees of the hospital (INCan 008/034/OMI) and that of the Faculty of Medicine at the Universidad Nacional Autónoma de México (UNAM-108/2015).

### Primary cell culture

Fresh samples of tumors were recovered from the operating room, collected in culture medium and immediately sent to cell culture facilities. The medium used for ovarian tumor cells was 1:1 Medium 105 (Merck, Darmstadt, Germany) and Medium-109 without phenol red (Thermo Fisher Scientific, Madison WI, USA) with 100 units/ml penicillin and 100 μg/ml streptomycin. Samples of the tumor (1 cm side) were minced in 2 mm fragments and then transferred to a solution of trypsin 0.25% in medium plus 0.1 mM EDTA (Ethane-1,2-diyldinitrilotetraacetic acid) (Merck) for 20 min. Isolated cells were recovered in 0.5% trypsin inhibitor (Merck) and 1.0% albumin in medium. Isolated cells were centrifuged at 400 g, counted and transferred to 75 cm^2^ culture dish (Thermo Fisher Scientific, Roskilde, Denmark) and incubated in medium plus 10% fetal bovine serum (FBS) (Thermo Fisher Scientific) at 37 °C in 5% of CO^2^ atmosphere in a humidified chamber incubator up to 80% of confluence. A successful sample was considered when the confluence was obtained within a week of culture. Confluent cells were collected from the culture dish by trypsin 0.1% in medium plus EDTA, rinsed two times with trypsin inhibitor by centrifugation. Cell viability was evaluated by trypan blue exclusion test (0.1%). Viable cells were seeded by triplicate in 48-well plates (Thermo Fisher Scientific) (10^4^ cell/well) in medium plus 1.0% charcoal dextran-treated FBS and treated after 24 h incubation, with 10^− 8^ M 17β-estradiol, testosterone, dihydrotestosterone and progesterone (Steraloids, Wilton NH, USA) reconstituted to a final 0.03% ethanol in medium for 60 h; control cells received the vehicle. At the end of the culture, cells were collected with trypsin 0.1% in medium plus EDTA in Eppendorff tubes, centrifuged at 400 g and resuspended in 0.1 ml of medium. Number of cells retrieved from triplicates of control and experimental groups were determined in a 4 mm^2^ area of a Neubauer camera. The mean value obtained for each treatment was normalized to the simultaneous control and considered as the value that corresponds to the tumor culture.

### Immunohistochemistry

A fragment of the ovarian tumor sample was processed for the detection of estrogen receptor alpha (ERα), progesterone receptor (PR) and androgen receptor (AR). Tissue microarrays (4 mm core) obtained from a representative region of paraformaldehyde fixed and paraffin embedded tumor samples, were sectioned at 3 μm thickness and placed on coated glass slides (Biocare Medical, Pacheco, CA, USA). Antigens were retrieved with Diva Decloaker (Biocare Medical) in a pressurized cooker at 110 °C for 10 min; endogenous peroxidase was blocked with Peroxidazed 1 (Biocare Medical). The slides were incubated overnight at 4 °C with the following polyclonal rabbit primary antibodies: anti-AR diluted 1:50 (Santa Cruz Biotechnology, Inc., Dallas, TX, USA), anti-ERα, 1:100 (Santa Cruz Biotechnology, Inc.), anti-PR, 1:250 (Cell Signaling Technology, Danvers, MA, USA), anti-cytokeratin [AE1/AE3 + 8/18], 1:100(Biocare Medical). The secondary antibody used was Mach2 anti-rabbit HRP (Biocare Medical). The tissue sections were counterstained with hematoxylin. The negative controls were sections in which the primary antibody was substituted with PBS. The positive control tissues (testis, endometrium and breast cancer) were as well included in each immune reaction. Positive reaction for AR, ER, and PR was detected through nuclear localization. The immunohistochemistry for hormone receptors were classified according to the immunoreactivity score (IRS) [27], a IRS ≥ 2.0 was considered positive. The samples were assessed in a double-blind protocol in three representative microscopic fields by two independent observers.

### Statistics

The frequency of positive reaction for each receptor in the histological subtypes was analyzed by Pearson chi-square. Data of number of cells recovered from the culture dishes were analyzed by one-way ANOVA. Comparisons between the values obtained between progesterone receptor positivity were evaluated by “t” Student’s test. Spearman’s correlation was calculated between cell number and IRS for progesterone receptor. *P* value of less than 0.05 being considered significant.

## Results

**Fig. 1:**
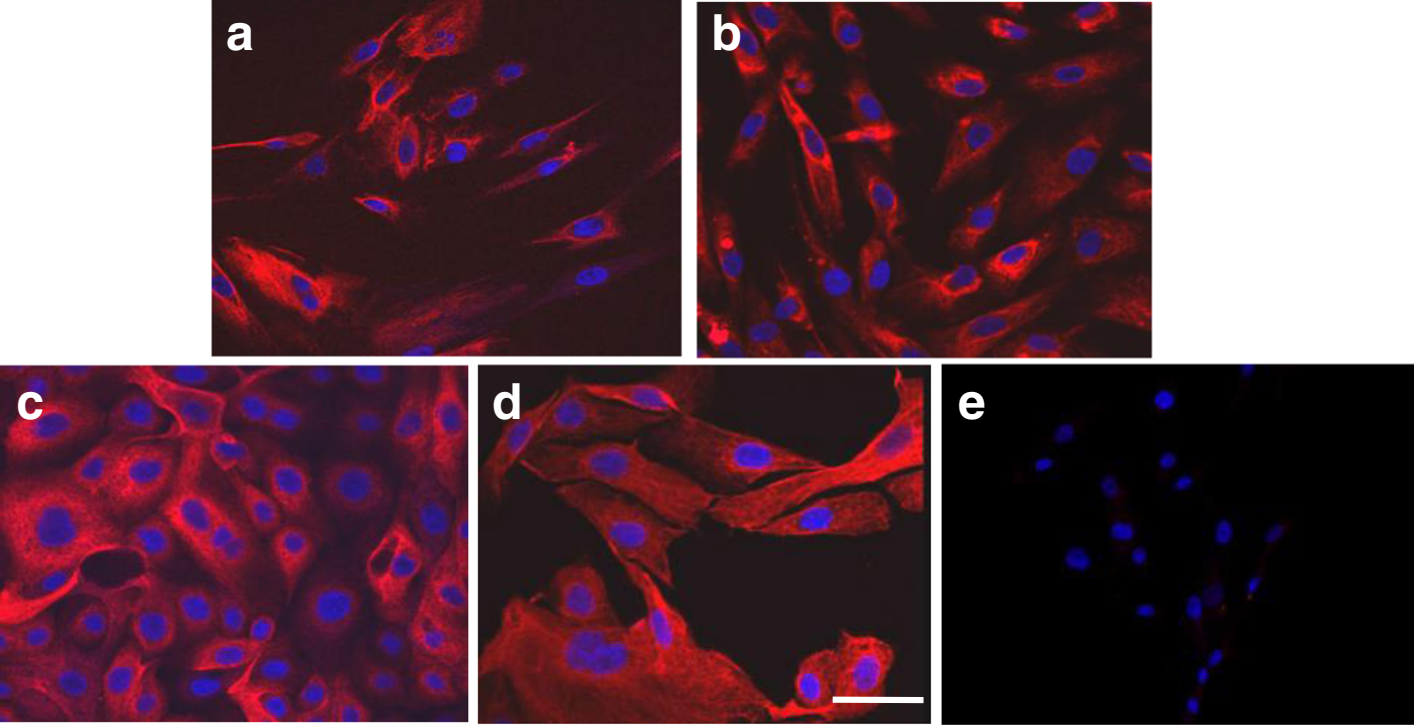
Primary culture of ovarian carcinoma cells. Immunofluorescence for cytokeratins of low-grade serous carcinoma, Pan Cytokeratin Plus antibody (red). Nuclei were stained with DAPI (blue). a) high grade serous carcinoma, b) endometrioid carcinoma, c) mucinous carcinoma, d) low grade serous carcinoma, e) negative control. Bar represents 12 μm

The total number of tumor samples processed to obtain cells for primary culture was obtained from 93 patients with ovarian carcinoma diagnosis, with only 74 of them being suitable for cell culture and 35/74 (47%) of the samples displayed a successful growth and reached the required confluence. The best results were obtained with LGSC 9/12 (75%) and the worst with HGSC 8/26 (31%); with a ratio of 15/27 (56%) of endometrioid subtype and 3/9 (33%) of mucinous carcinoma. The culture medium selected (105–109, 1:1) favored epithelial cell survival and growth and at the end of the culture 70% of the cells showed an epithelial phenotype and expressed cytokeratins positivity, as shown in Fig. 1.

The characteristics of the patients that were evaluated ex vivo through a primary cell culture of the ovarian tumor (*n* = 35) are summarized in Table 1.

**Table 1:** Characteristics of patients according to histological subtypes of ovarian carcinoma

	HGSC	Endometrioid	Mucinous	LGSC
Median age (years)	49	47	54	40
Menopause	3/8	5/15	3/3	3/9
Oral contraceptives	None	2/15	None	None
FIGO stages				
I	–	7/15	3/3	4/9
II	2/8	1/15	–	–
III	4/8	6/15	–	5/9
IV	2/8	1/15	–	–
Histological grade				
G1		1/15		
G2		11/15		
G3		3/15		
Debulking				
Optimal < 1 cm	8/8	12/15	3/3	8/9
Suboptimal > 1 cm	–	3/15	–	1/9

*HGSC*: high grade serous carcinoma, *LGSC*: low grade serous carcinoma

**Fig. 2:**
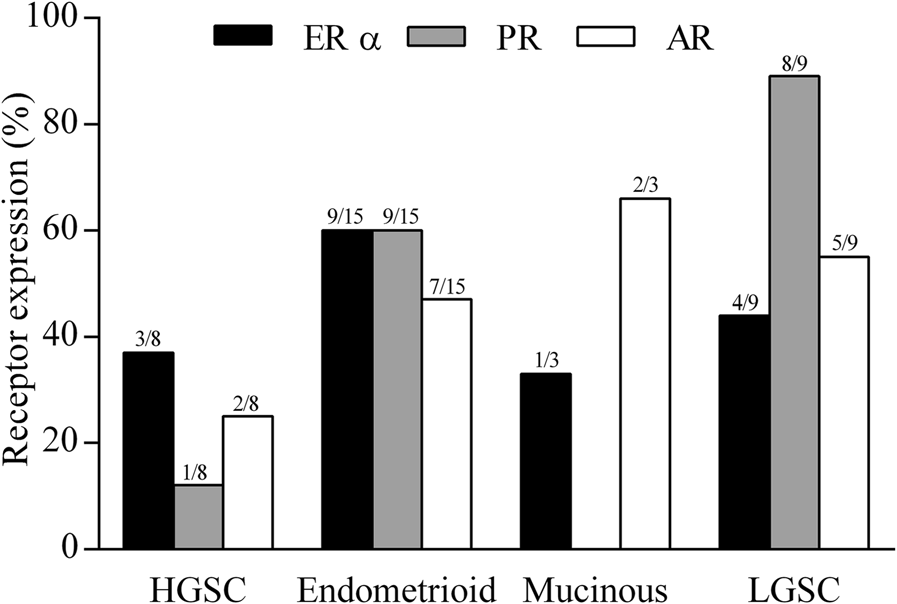
Frequency of expression of steroid hormone receptor. Positive reactions for estrogen receptor α (ERα), progesterone receptor (PR) and androgen receptor (AR) in ovarian carcinomas stratified inhigh grade serous carcinoma (HGSC), endometrioid, mucinous and low grade serous carcinoma (LGSC) subtypes

The frequency of the presence of steroid hormone receptor in the tumor samples previous to the primary culture in the histological subtypes is shown in Fig. 2.

The relative number of cells recovered at the end of the culture after treatment with E_2_, P_4_, T and DHT is shown in Table 2.

**Table 2:** Relative cell number (treated/control) after steroid hormone treatment in histological subtypes of ovarian carcinoma

Treatment	LGSC	n	HGSC	n	Endometrioid	n	Mucinous	n	*P*
Estradiol	126 ± 37	9	103 ± 52	8	93 ± 32	15	109 ± 35	3	0.118
Progesterone	114 ± 34	8	116 ± 37	8	68 ± 20	14	86 ± 25	3	**0.002**
Testosterone	108 ± 41	9	126 ± 51	7	88 ± 22	14	98 ± 19	3	0.137
DHT	106 ± 26	8	122 ± 37	8	105 ± 40	13	82 ± 19	2	0.529

Values indicate mean ± SD, *P* values of one-way ANOVA

**Fig. 3:**
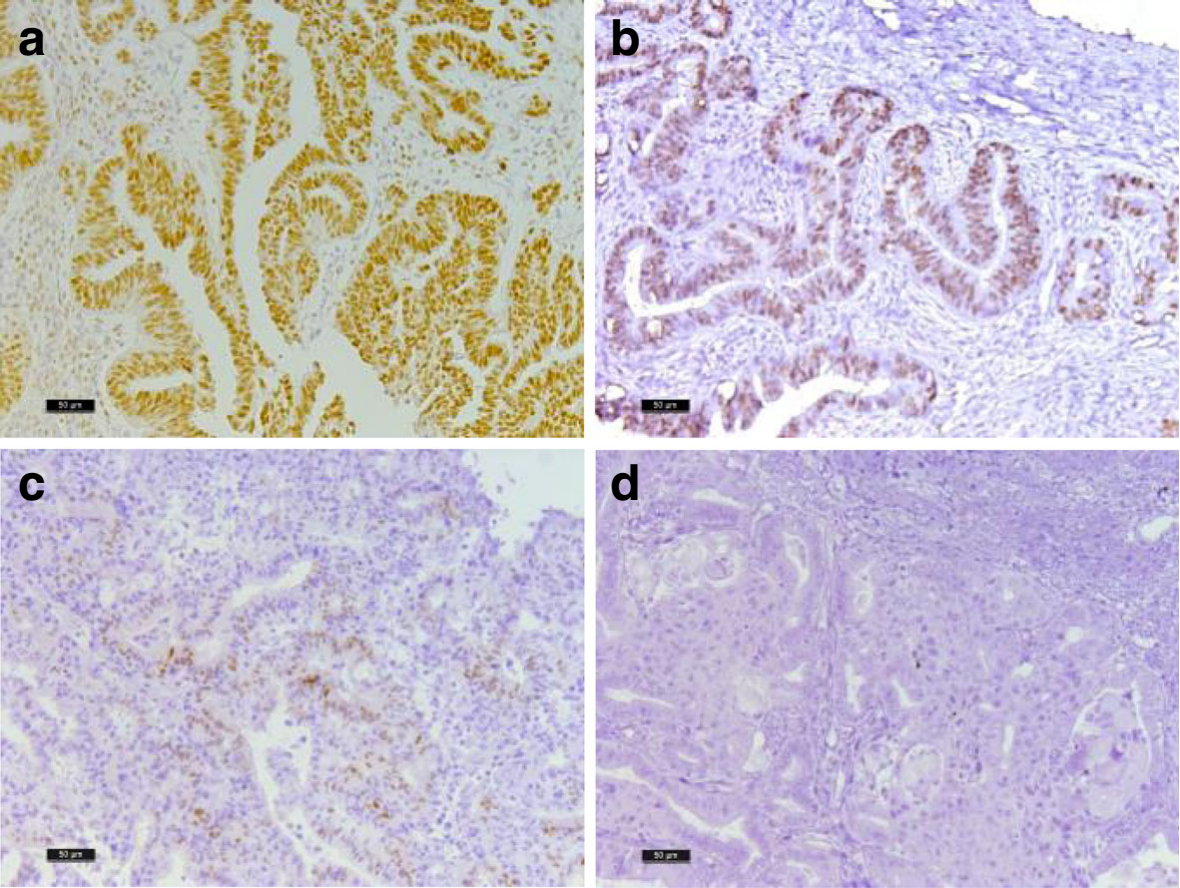
Immunohistochemistry images of progesterone receptor in endometrioid ovarian carcinoma. a-c) different levels of progesterone receptor positivity are observed between patient samples. d) negative control. Bar represents 50 μm

**Fig. 4:**
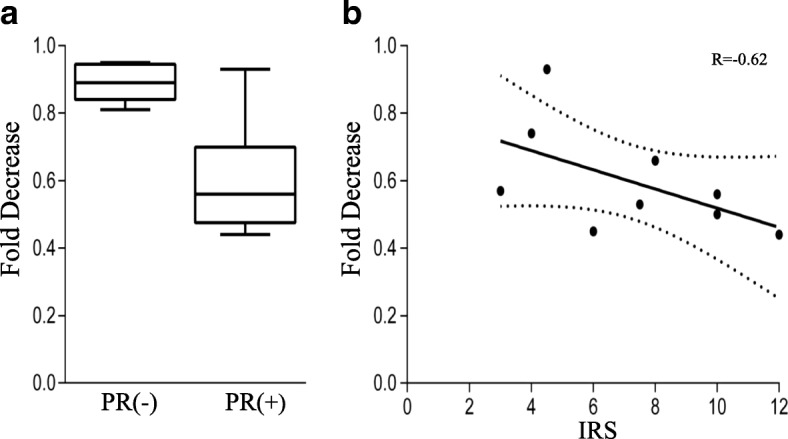
Relative cell number in primary culture of ovarian carcinoma. Fold decrease from simultaneous control in the number of cells recovered at the end of primary culture of endometrioid ovarian carcinoma treated with 10^− 8^ M progesterone. A) Values of samples separated by the presence of progesterone receptor, PR (−) *n* = 5, PR (+) *n* = 9, *P* = 0.002. B) Spearman’s correlation between relative cell number in PR (+) samples and the immunoreactive score (IRS) of each sample. R = − 0.62, *P* = 0.07

A significant reduction (32%) in cell number was observed in endometrioid ovarian carcinoma with progesterone treatment. On the other hand, a possible increase in cell number by E_2_ in LGSC, and for T and DHT in HGSC (*P* = 0.12 and 0.14, respectively) should be analyzed in a larger number of patients. Considering the presence of PR in the endometrioid ovarian carcinoma, samples that were PR (+) displayed a significant reduction in cell number compared with that of the PR (−) tumors, Figs. 3 and 4a. Tumor samples positive for progesterone receptor were further analyzed, a correlation between the relative cell number and IRS for PR (+) samples resulted in a value of R = − 0.62 with *P* = 0.075, Fig. 4b. No differences in the relative cell number were observed in samples treated with E_2_, T and DHT separated by the presence of ERα and AR in the tumor (data not shown).

## Discussion

The efficiency of the growth in culture (47%) was similar to that obtained in previous reports for primary cultures of ovarian carcinoma [28]. The steroid hormone treatment was applied in a medium with low serum level and the presence of endogenous steroids was minimized by the use of charcoal dextran-treated fetal bovine serum. There was variation between the tumor cultures, and the triplicate measurement of the experimental group was normalized to the simultaneous control and considered as the value corresponding to each tumor. The final relative cell number measured at the end of the culture is a balance between proliferation and cell death. Results did not provide information about cell division and apoptosis during the 60 h of steroid hormone treatment.

The frequency of detection of AR, ERα and PR through immunohistochemistry and the immunoreactive score is an estimation of the receptor presence and constitutes a reliable indicator about the endocrine profile of the tumor. The changes induced by a treatment with the steroid hormones provide information about the importance of the sexual steroid hormones in malignancy progression. Primary cultures closely resemble the physiology of cells in vivo, considering the histological subtype of the carcinoma, which leads us to propose that the ex vivo evaluation of the hormonal response, along with the receptor profile of the tumor will allow a better characterization of each patient’s carcinoma.

Progesterone treatment significantly reduced the relative number of cultured cells and this effect was observed only in endometrioid ovarian carcinoma, with this reduction being greater (43%) when only endometrioid carcinoma PR (+) was considered. Moreover, a relationship between the IRS for PR and the degree of cell number reduction seems to be present. Previous studies have provided evidence that progesterone treatment reduces cell proliferation and induces apoptosis in human ovarian carcinoma cell lines [20, 21]. Moreover, progesterone induces apoptosis by activating caspase-8 and caspase-3 in OVCA cell lines [22]. Therefore, the reduction in the cell number observed in the culture of endometrioid carcinoma by progesterone treatment was probably caused by a reduction in cell division, by an increase of apoptosis or both. The values obtained with E2, T and DHT were not significant, although an effect of these steroid hormones cannot be dismissed until a more extensive study is carried out.

The presence of progesterone receptor has been previously associated to better clinical outcome in patients with ovarian carcinoma [13–15]. Similarly, a large multicenter study showed a better outcome associated to progesterone receptor in endometrioid and HGSC subtypes [12]. The endometrioid subtype of ovarian carcinoma has the highest frequency of estrogen receptor as well as a high percentage of triple positive profile [29] suggesting that endometrioid subtype could be a hormone sensitive carcinoma. Interestingly, endometrioid ovarian carcinoma seems to preserve characteristics of endometrial cells, in which the anti-proliferative effect of progesterone is evident both in normal and cancer cells [30].

There is evidence that the use of oral contraceptives and pregnancy are protective factors for ovarian cancer incidence [31]. Progestins are the main component of contraceptives and the highest levels of progesterone are reached during pregnancy. Then, an anti-proliferative or a pro-apoptotic effect of progesterone on pre-cancerous lesions has been proposed [32, 33]. After menopause, progesterone is found in negligible amounts in serum [34], which implies that the protection of progestin would be absent in postmenopausal women, when the incidence of ovarian cancer increases. Another important issue to be considered is the complex cross-talk between the steroid hormones themselves and steroid receptors; for example, progesterone induces changes in ERα chromatin binding events in breast cancer cell lines (MCF-7 and T-47D) and leads new gene expression profiles that are associated to a better prognosis in breast cancer patients; moreover, P4 inhibits estrogen proliferative effect in primary culture of breast tumor tissue and xenografts of tumor cells in mice [35].

## Conclusions

The evidence provided by the present study sustains the importance of progesterone in the reduction of ovarian cancer progression. The effect is evident in endometrioid histological subtype and it is related to the presence of progesterone receptor in the ovarian carcinoma. Present results, together with previous relevant studies, support that epithelial ovarian cancer is a hormone responsive tumor and open the possibility to consider individualized treatments of the malignancy.

## Abbreviations

AR: Androgen receptor
DHT: Dihydrotestosterone
E_2_: 17β-estradiol
EDTA: Ethane-1,2-diyldinitrilotetraaceticacid
ERα: Estrogen receptor alpha
HGSC: High grade serous carcinoma,
IRS: Immunoreactivity score
LGSC: Low grade serous carcinoma
P_4_: Progesterone
PR: Progesterone receptor
T: Testosterone
